# Exosomes derived from plasma: promising immunomodulatory agents for promoting angiogenesis to treat radiation-induced vascular dysfunction

**DOI:** 10.7717/peerj.11147

**Published:** 2021-04-02

**Authors:** Yanxi Li, Ping Lyu, Yiting Ze, Peiran Li, Xinyi Zeng, Yixin Shi, Bingrun Qiu, Ping Gong, Yang Yao

**Affiliations:** State Key Laboratory of Oral Diseases, West China Hospital of Stomatology, Sichuan University, Chengdu, Sichuan, China

**Keywords:** Plasma exosomes, Angiogenesis, Ionizing radiation, Immunity, Tissue regeneration, Macrophages

## Abstract

Ionizing radiation (IR)-induced vascular disorders slow down tissue regeneration. Exosomes derived from plasma exhibit potential to promote angiogenesis; meanwhile, the immune microenvironment plays a significant role in the process. This study aimed to test the hypothesis that plasma exosomes promote angiogenesis in irradiated tissue by mediating the immune microenvironment. First, we explored the impact of IR on macrophages. We found that cell viability and capacity for promoting angiogenesis were decreased in irradiated macrophages compared to control macrophages. Then, we isolated and characterized rat plasma-derived exosomes (RP-Exos) which were defined as 40–160 nm extracellular vesicles extracted from rat plasma. Afterward, we evaluated the effects of RP-Exos on the behaviors of irradiated macrophages. Our results show that RP-Exos promoted cell proliferation. More importantly, we found that RP-Exos stimulated the immune microenvironment in a manner that improved the angiogenesis-related genes and proteins of irradiated macrophages. The supernatant of macrophage cell cultures was used as conditioned medium to treat human primary umbilical vein endothelial cells, further confirming the pro-angiogenic ability of macrophages receiving RP-Exo intervention. RP-Exos were used in vivo to treat irradiated skin or calvarial defects in irradiated Sprague-Dawley male rats. The results indicated the ability of RP-Exos to enhance angiogenesis and promote tissue regeneration. Our research suggested the potential of plasma exosomes to be used as immunomodulatory agents with angiogenic capacity to treat radiation-associated vascular disorders and facilitate tissue repair.

## Introduction

Ionizing radiation (IR) is one of the most effective ways to control various types of tumors, but it can cause side effects, including impaired wound healing and radiation bone injury (Cox & Dyson, 1995; Dormand, Banwell & Goodacre, 2005; Du et al., 2020). Vascular dysfunction is one of the most significant manifestations of irradiation-mediated structural disorganizations (Guipaud et al., 2018; Lindblom et al., 2019; Soloviev & Kizub, 2019). To alleviate such side effects, intensive research efforts have focused on finding different biological agents with the potential to promote angiogenesis (Mashiko et al., 2018; Olascoaga et al., 2008).

Exosomes are 40–160 nm nano-sized particles, usually defined as endosomal origin extracellular vesicles (Im et al., 2019; Kalluri & LeBleu, 2020; Liao et al., 2019). They can be released from any cell type and are enriched in body fluids such as plasma, saliva, and urine (Kowal, Tkach & Théry, 2014). Previous studies have found that exosomes contain functional mRNAs and small noncoding RNAs, constituting an intercellular passage of communication (Ludwig & Giebel, 2012; Simons & Raposo, 2009; Yao et al., 2018).

Many studies focus on the therapeutic use of exosomes isolated from cell culture media, but this application is limited by the complicated isolation and culture process of primary cells, inadequate production, and the difficulty in controlling functional stability and biosafety. In contrast, exosomes extracted from plasma are safer and more convenient to access. It is also easier to control their production yield.

Several studies have applied exosomes of different origins to wounds to accelerate wound healing (Zhang et al., 2015; Zhao et al., 2017). The application of blood plasma exosomes for cardioprotection has recently received heightened attention (Davidson et al., 2018; Vicencio et al., 2015). In particular, exosomes derived from platelet-rich plasma (Guo et al., 2017) and umbilical cord blood plasma (Hu et al., 2018b) enhance angiogenesis and promote wound healing. However, the role of the immune system in this process is not clear.

As the first line of defense confronting foreign bodies, macrophages comprise a crucial component of the innate immune system. Monocytes and macrophages play a pivotal role in local neovascularization by secreting pro-inflammatory and pro-angiogenesis cytokines as well as stimulating the expression of vascular endothelial growth factor (VEGF) receptors (Kir et al., 2018; Ribatti & Crivellato, 2009; Varricchi et al., 2018). Considering that exosomes can mediate the immune response positively or negatively (Robbins & Morelli, 2014), we investigated how rat plasma exosomes (RP-Exos) modulate the immune response of irradiated macrophages and how this modulation affects the vascular formation process.

**Figure 1:**
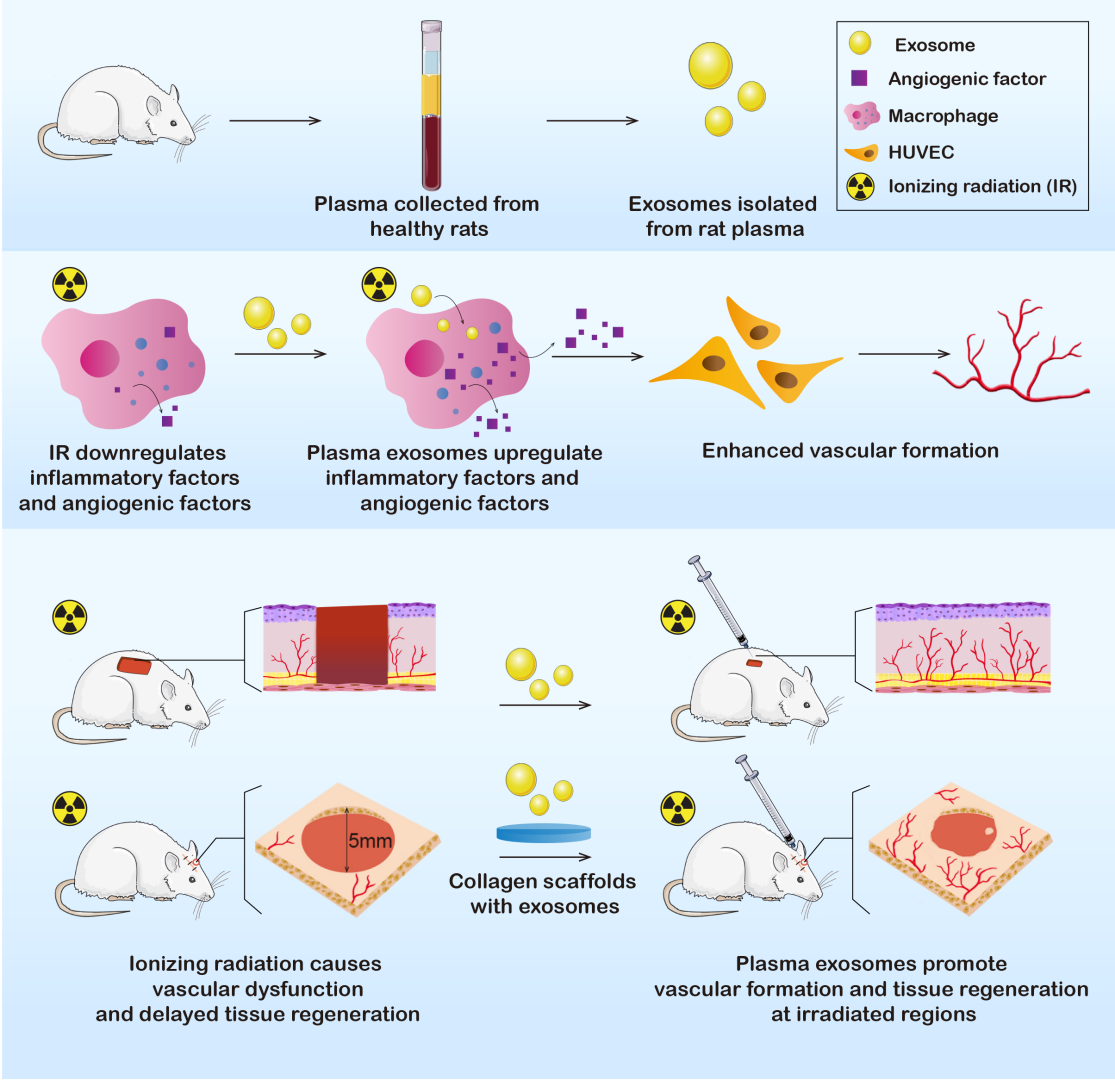
Exosomes were derived from healthy rat plasma. RP-Exos stimulated inflammatory reactivity of macrophages, which further enhanced vascular formation of HUVEC cells by upregulating angiogenic factors. Afterward, RP-Exos were applied to in vivo experiments, where they promoted angiogenesis and tissue regeneration in both irradiated skin and bone defects.

In this study, we extracted plasma exosomes from healthy Sprague-Dawley (SD) rats. Exosomes were further applied in vitro and in vivo to determine their effects on immunoreactions and angiogenesis. The results showed that the RP-Exos stimulated the immune environment in a manner that improved vascular formation, indicating the potential to promote wound healing after irradiation and to cure radiation-induced tissue injury (Fig. 1).

## Materials & Methods

### Cell culture

The murine macrophage cell line RAW 264.7 and human primary umbilical vein endothelial cells (HUVECs) were purchased from American Type Culture Collection (ATCC, USA). RAW 264.7 cells were cultivated in Dulbecco’s Modified Eagle Medium (DMEM; Gibco, USA) supplemented with 10% fetal bovine serum (FBS; Gibco) and Penicillin-Streptomycin Solution (100 IU/ml penicillin, 100 µg/mL streptomycin; Hyclone, USA). HUVECs were cultivated in Endothelial Cell Growth Medium (EGM; Lonza, Switzerland). Cells were cultured at 37 °C in a 5% CO_2_ humidified incubator. Cell medium was changed every 2 days. Cells were passaged using scrapers (for RAW 264.7) or trypsin solution (for HUVECs) when the culture reached approximately 80% confluence.

RAW 264.7 cells were cultured for 2 days and reached 30% confluence. Afterwards, they were divided into 3 treatment groups: (1) E0R0 group, receiving neither RP-Exos nor radiation treatment; (2) E1R1 group, receiving 2 Gy radiation and 400 µg/ml RP-Exos treatment for 3 days; and (3) E0R1 group, receiving 2 Gy radiation and treatment with an equal volume of PBS for 3 days. Radiation intervention was performed in the Chengdu Seventh People’s Hospital, China.

The supernatant of RAW 264.7 cells from the three groups was harvested, and then filtered through a 0.22 µm hydrophilic Polyethersulfone (PES) membrane (Millipore, USA) to exclude macrophages. Then, the filtrate was stored at −80 °C to be used as conditioned medium (CM) to treat HUVECs.

### Isolation and identification of rat plasma exosomes

Platelet-free rat plasma was collected from 5 Eight-week-old SD male rats. Briefly, rats were anesthetized by injecting chloral hydrate (300 mg/kg) intraperitoneally. Fresh blood was obtained via the abdominal aorta using EDTA-containing tubes. Plasma was collected by centrifuging blood at 1500 × g for 20 min at 20 °C. Then, plasma was further centrifuged at 2000 × g for 30 min at 20 °C to remove platelets.

Exosomes were isolated from rat plasma using Exoquick Exosome Isolation Kit (System Biosciences, USA) following kit guidelines. The concentration was quantified using a bicinchoninic acid (BCA) assay according to the manufacturer’s protocol (Beyotime, China). The morphology of RP-Exos was observed using a transmission electron microscope (TEM, Hillsboro, USA). RP-Exo size was detected by nanoparticle tracking analysis (NTA; NanoSight 300, Malvern Panalytical, China) and the expression of exosome markers was evaluated by western blot analysis.

### Uptake of RP-Exos by macrophages in vitro

RP-Exos were fluorescently labeled using Dil (Beyotime), for 10 min. Afterward, RP-Exos were diluted to 400 µg/ml and added to the RAW 264.7 cells and incubated for 24 h. The cells were washed 3 times with PBS, and stained with 4, 6-diamidino-2-phenylindole (DAPI, Solarbio, China) for 10 min. Fluorescence microscopy (Leica, Germany) was used to visualize the endocytosis of RP-Exos by RAW 264.7 cells.

### Cell viability assay and cell cycle analysis

The viability of RAW 264.7 cells was detected by a Cell Counting Kit-8 (CCK-8) assay (Dojindo, Japan) at 0, 1, 3, and 5 days. The number of viable cells was determined by measuring the optical density (OD) value at 450 nm in 5 wells per group using a microplate spectrophotometer (Thermo Fisher Scientific, USA).

For cell cycle analysis, RAW 264.7 cells of the three groups were collected and fixed in a 70% ethyl alcohol solution at 4 °C overnight. Afterwards, cells were stained with 10% RNase A and 90% propidium iodide solution (Keygen Biotech, China) for 30 min. Fluorescence intensity at 488 nm was estimated using a flow cytometer (Attune Nxt, Invitrogen, USA). The cell cycle distribution was analyzed with the Modfit LT software.

### Transcriptome sequencing and analysis

Total RNA was extracted from RAW 264.7 cells using TRIzol reagent (Invitrogen). The purity and concentration of RNA were tested (NanoPhotometer, Implen, Germany). RNA integrity was evaluated with the Agilent 2100 Bioanalyzer (Agilent Technologies, USA). Libraries were constructed using the TruSeq Stranded mRNA LT Sample Prep Kit (Illumina, USA). The libraries were sequenced on an Illumina HiSeq X Ten platform, and 150 bp paired-end reads were generated. Raw data were first processed using Trimmomatic. About 85.80 G clean reads for each sample were obtained for downstream analyses. Differential expression analysis was performed using the DESeq (2012) R package. Significantly differential expression was defined as *P* value <0.05 and foldchange >2 or <0.5. Gene Otology (GO) enrichment and Kyoto Encyclopedia of Genes and Genomes (KEGG) pathway enrichment analysis of DEGs were carried out. All of the above procedures were conducted by OE Biotech Co., Ltd. (Shanghai, China).

### Quantitative RT-PCR

Total RNA was extracted as described above. RNA was reverse transcribed into cDNA using the PrimeScript RT Reagent Kit (Takara, Japan). The expression of genes was measured using the SYBR Premix Ex Taq II Kit with QuantStudio 7 Flex system (Applied Biosystems, Thermo Fisher Scientific). Primers for the target genes are presented in Table S1.

### Western blot

Total protein was extracted using the Total Protein Extraction Kit (SAB, USA) according to the manufacturer’s instructions. Protein concentration was quantified using a BCA protein assay (Beyotime). Sixty micrograms of total protein were separated by 10% sodium dodecyl sulphate–polyacrylamide gel electrophoresis and transferred to 0.22 µm polyvinylidene difluoride (PVDF) membranes at 200 mA for 1 h. The membranes were blocked with 10% albumin bovine (BSA, Biofroxx, Germany) for 1 h at room temperature. The blots were probed with primary antibodies for 24 h at 4 °C and further incubated with secondary antibodies for 1 h at room temperature. These primary antibodies included CD63 (System Biosciences, 1:1000), TSG101 (System Biosciences, 1:1000), ADM (Santa Cruz Biotechnology, USA, 1:1000), VEGFA (Santa Cruz Biotechnology, 1:1000), and NDRG1 (Santa Cruz Biotechnology, 1:1000). The supernatant of RP-Exos extract was used as a negative control for exosome characterization.

### Tube formation assay

To promote endothelial tube formation, 100 µl chilled Matrigel matrix (5 mg/ml, BD Biosciences, USA) was tilted on each well of a 48-well plate. After polymerization, HUVECs suspended in CM were seeded onto the Matrigel with 180,000 cells per well. HUVECs were also divided into 3 groups according to the CM they received. CM for the E0R0 group came from the supernatant of RAW 264.7 cells in the E0R0 group, and so on. Images were taken after 4, 8, and 12 h. Tubes were analyzed using ImageJ software based on total tube length and numbers of nodes, junctions, and branches.

### Synthesis of Exo/PBS collagen scaffolds

Rat tail collagen I (3 mg/ml, BD Biosciences) hydrogels were prepared to load either Exo or PBS. For each scaffold, 800 µg Exo or equal volume of PBS and 100 µl hydrogel solution were added to each well of a 384-well plate. The mix was incubated at 37 °C for 30 min to promote consolidation.

### Animal care and radiation treatment

All animal experiments were approved by Research Ethics Committee of West China Hospital of Stomatology, Sichuan University (WCHSIRB-D-2017-050). SD male rats were purchased from Chengdu Dossy Biological Technology Co., Ltd and housed in the animal center of West China Hospital of Stomatology in keeping with national standard *Laboratory Animal-Requirements of Environment and Housing Facilities* (GB 14925-2001). No enrichment was provided in this study. Food or water intake, and activity of rats were monitored once daily. Eighteen rats were recruited in animal experiments. Dislocation of cervical vertebra was applied to euthanize rats within limited pain. The rats were also euthanized if they didn’t incline to feed or drink. All the animal experiment procedures were conducted by same investigators under similar situation. Immediately after surgery, intravenous injection of penicillin (20,000 units) per rat was performed. The operators who completed surgical procedures or data analyses did not know the group allocation.

Radiation treatment was performed in the Seventh People’s Hospital of Chengdu, China.For the skin defect experiment, whole rat bodies were irradiated with a single dose of 6 Gy. For the bone defect experiment, a square area (width = three cm) at the center of the rat’s cranial bone was locally irradiated with a single dose of 15 Gy.

### Defects creation and RP-Exos treatment

For the skin defect experiment, six SD rats were randomly (Microsoft Excel was used to generate random numbers) divided into two groups: (1) E0R1 group, in which irradiated rats were injected with PBS solution; or (2) E1R1 group, in which irradiated rats were treated with RP-Exos solution. Briefly, 4 weeks after radiation, two square, full-thickness (2 cm ×2 cm) wounds were created on each side of the rat dorsal skin. In the E0R1 group, 200 µl PBS solution was injected around each wound, while in the E1R1 group, 200 µg RP-Exos dissolved in 200 µl PBS was injected. Injections were carried out every 2 days. Twenty-one days after surgery, all rats were euthanized and the skin in the target areas was obtained. Each wound area was regarded as an experimental unit, with 6 units in each group.

For the bone defect experiment, twelve SD rats were also randomly assigned to two groups, the E0R1 group or the E1R1 group. One week after radiation, two 5-mm diameter defects were made in each rat at the center of cranial bone using a trephine drill. In the E1R1 group, hydrogels with 800 µg RP-Exos (immediately after surgery, one time only) and RP-Exos solution with 80 mg Exos (subcutaneous injection every 4 days after surgery, 14 times total) were used. In the E0R1 group, the hydrogel contained equal volume of PBS and PBS solution was used as the negative control. Eight weeks after surgery, all rats were sacrificed to obtain the target calvarial bone. A bone defect area was regarded as an experimental unit, with 12 units in each group. All target samples were fixed in 4% paraformaldehyde for 5 days.

### Micro-CT

The cranial specimens were analyzed using a micro-CT scanner (SCANCO, Switzerland). Three-dimensional (3D) reconstruction of the images was carried out to evaluate new bone formation in the defects, as well as trabecular number (Tb.N, 1/mm), and trabecular separation (Tb.Sp, mm).

### Histology

All skin specimens were dehydrated and subsequently embedded in paraffin. For bone specimens, a decalcification process was added before dehydration. After sectioning the tissues, hematoxylin & eosin (HE) staining or Masson staining was performed. For angiogenesis analysis, immunohistochemical and immunofluorescence staining were carried out with the following primary antibodies: rabbit anti-CD31 (1:2000, Servicebio, China) and rabbit anti-αSMA (1:2000, Boster Biological Technology, USA). Target fields from each staining sample were recorded.

### Statistics

Statistics were collated using Microsoft Excel. Data analyses were carried out using GraphPad Prism. Normality test and homogeneity of variance analysis were performed at first to decide the statistical approach. Comparisons between two groups were analyzed using a two-tailed t test, and one-way ANOVA analysis was used to compare all three studied groups, only if the data were normally distributed and variances were similar among different groups. Otherwise, recommended statistical methods were used. Data were presented as the mean ± standard deviation. *P* values <0.05 were considered significant (* = *P* < 0.05, ** =*P* < 0.01, *** = *P* < 0.001, ***** = *P* < 0.0001).

## Results

### Characterization and endocytosis of RP-Exos

**Figure 2:**
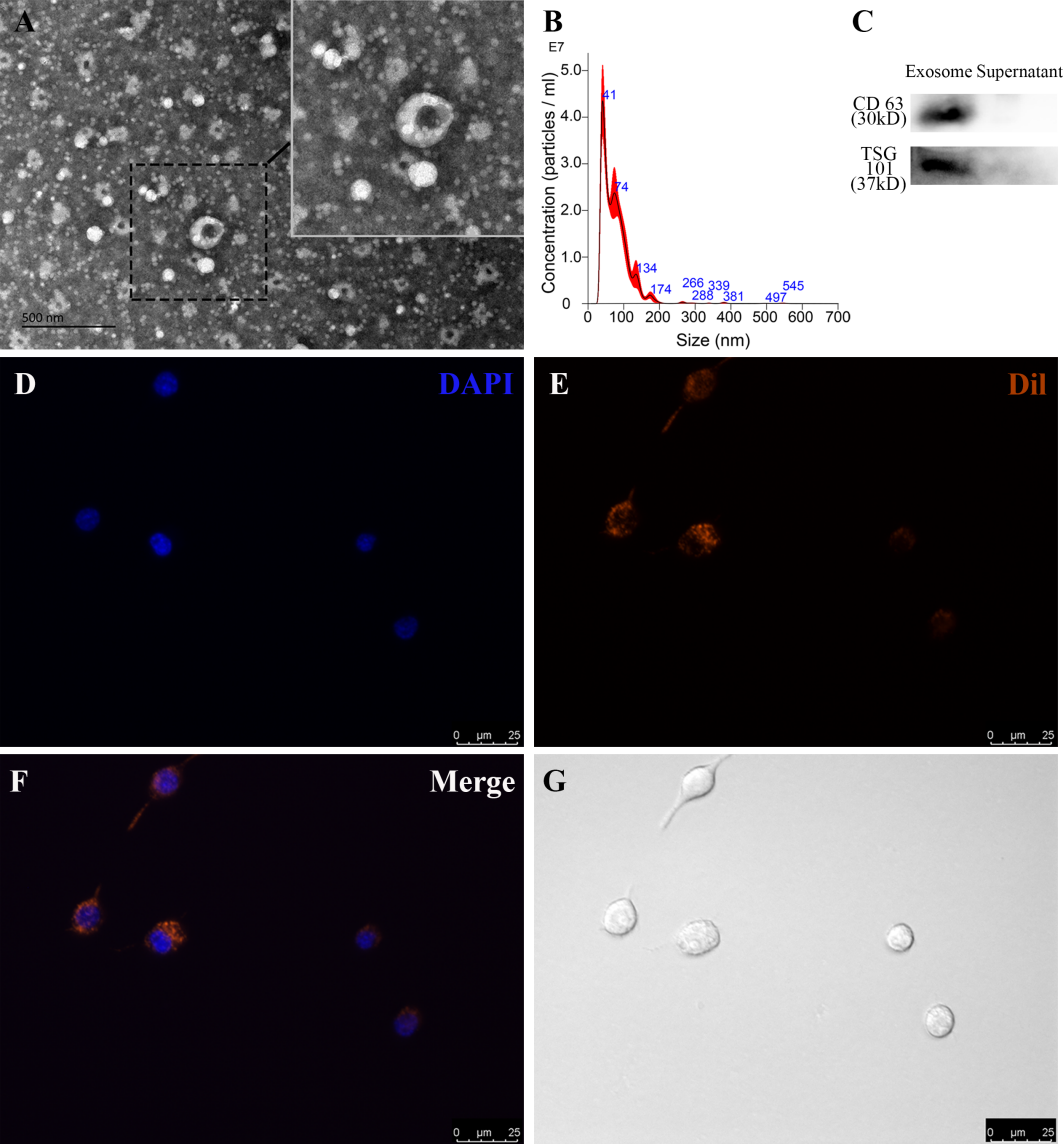
Characterization and endocytosis of RP-Exos. (A) Morphology of RP-Exos observed by transmission electron microscope (TEM). Scale bar = 500 nm. (B) Particle diameter distribution of RP-Exos measured by nanoparticle tracking analysis (NTA). (C) Western blot analysis of CD63 and TSG101 on RP-Exos and supernatant. (D–G) Representative fluorescence and bright-field images of RP-Exos endocytosed by macrophages. Nuclei of macrophages were stained with DAPI (blue). RP-Exos were stained with Dil (red). Bars = 25 um.

To identify RP-Exos, NTA, TEM, and western blot were performed. The average particle diameter of the samples was 76.7 nm (Fig. 2B). Typical cup-shaped morphology of RP-Exos was observed in the TEM image (Fig. 2A). Western blot analysis confirmed the expression of exosome markers CD63 and TSG101 in the samples (Fig. 2C). The endocytosis of RP-Exos was observed through fluorescence staining. As shown in the Figs. 2D–2G, RP-Exos (red fluorescence) were detected in the cytoplasm of RAW 264.7 cells.

### IR inhibited cell viability and pro-angiogenic function in macrophages

**Figure 3:**
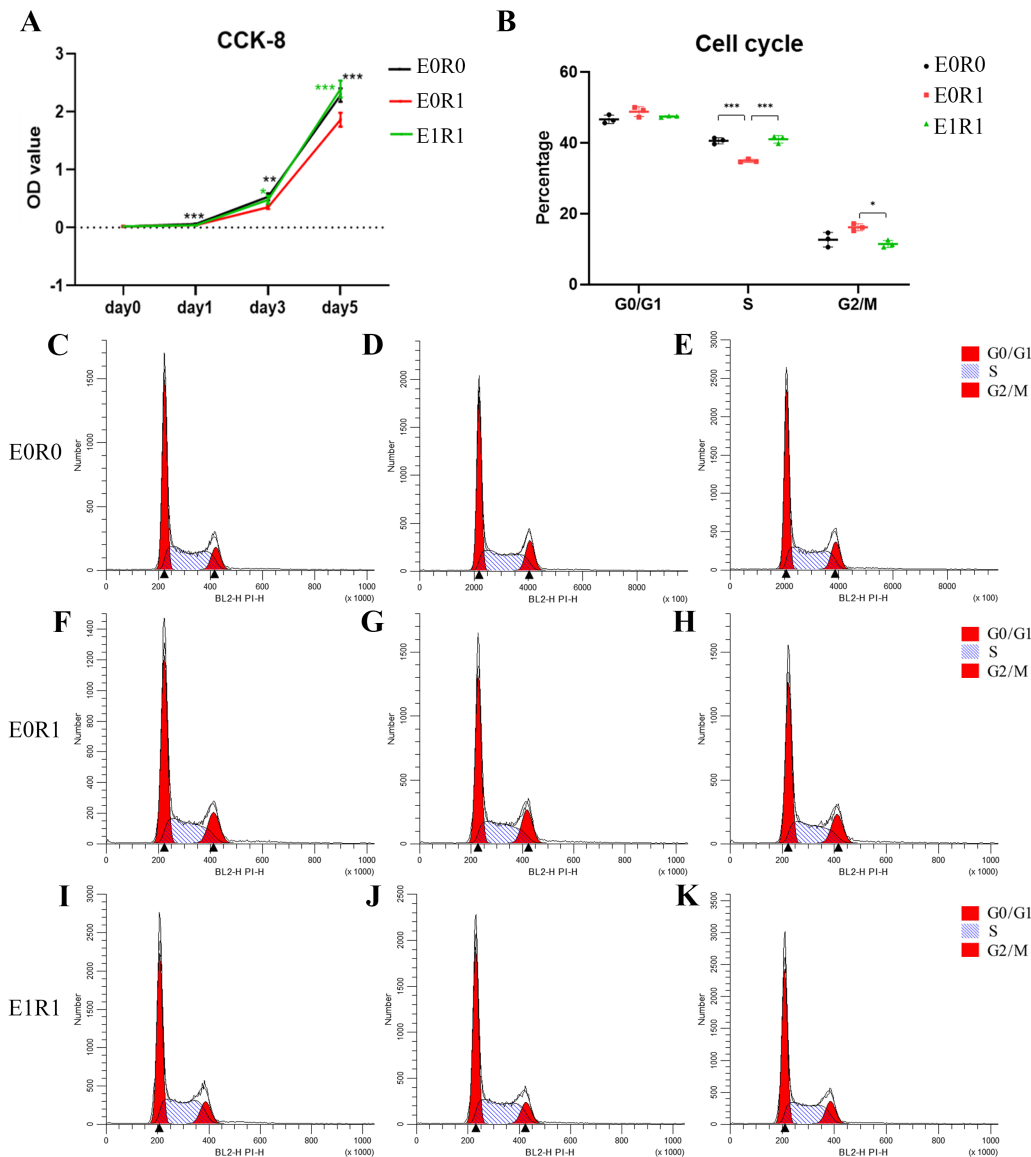
RP-Exo reversed IR effects on cell viability and cell cycle distribution of macrophages. (A) Cell viability as assessed by CCK-8 assay. Ionizing radiation impaired proliferation of macrophages while RP-Exos rescued the adverse effect. (B) Quantitative analysis of cell cycle distribution, *n* = 3 per group. IR induced macrophage cell cycle to arrest in the G2/M phase while RP-Exos inhibited the adverse effect. E0R0: untreated macrophages. E0R1: macrophages receiving ironizing radiation. E1R1: macrophages receiving ironizing radiation and RP-Exo intervention. * = *P* < 0.05, ^∗∗^ = *P* < 0.01, ^∗∗∗^ = *P* < 0.001. (C–K) Cell cycle distribution of macrophages in different groups.

The results of the CCK-8 assay indicated significantly decreased viability of macrophages after receiving 2 Gy radiation (Fig. 3A). As the cell cycle analysis showed (Figs. 3B, 3C–3K, radiation treatment induced arrest in the G2/M phase, a result that agrees with previous studies (Miyata et al., 2001). We propose that G2/M phase arrest is a possible mechanism by which IR inhibits cell growth.

**Figure 4:**
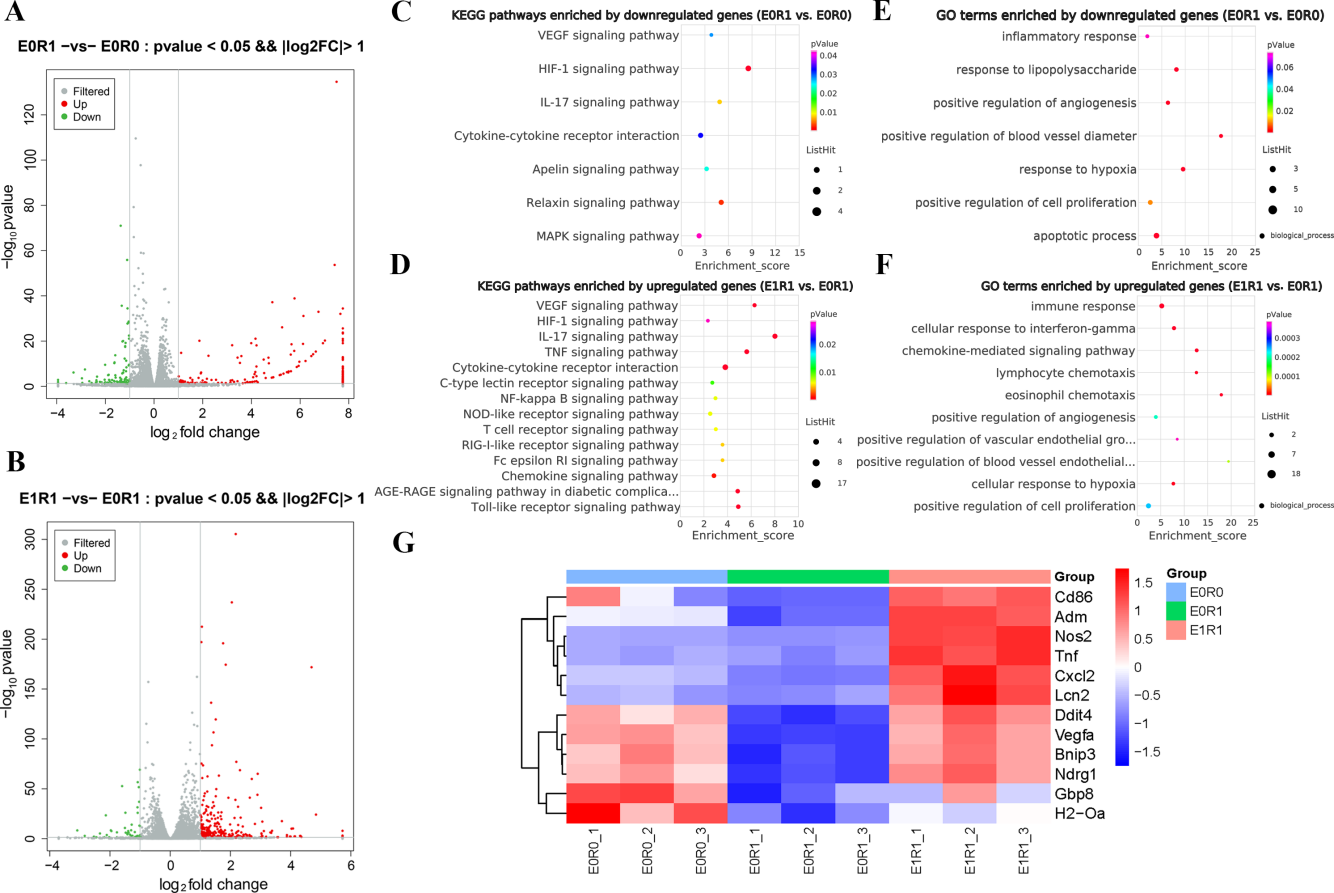
RP-Exos promoted macrophage inflammatory and pro-angiogenic functions, which were decreased by IR. (A–B) Differentially expressed genes shown in a volcano plot (E0R0 vs E0R1; E1R1 vs E0R1). (C, E) KEGG and GO analysis. The downregulated genes in the E0R1 group compared with the E0R0 group were enriched in inflammatory and pro-angiogenic pathways or GO terms. (D, F) KEGG and GO analysis. The upregulated genes in E1R1 group compared with E0R1 group were also enriched in inflammation and angiogenesis-related pathways or GO terms. (G) Heat map analysis of candidate genes. E0R0: normal macrophages. E0R1: macrophages receiving ironizing radiation. E1R1: macrophages receiving ironizing radiation and RP-Exo intervention.

**Figure 5:**
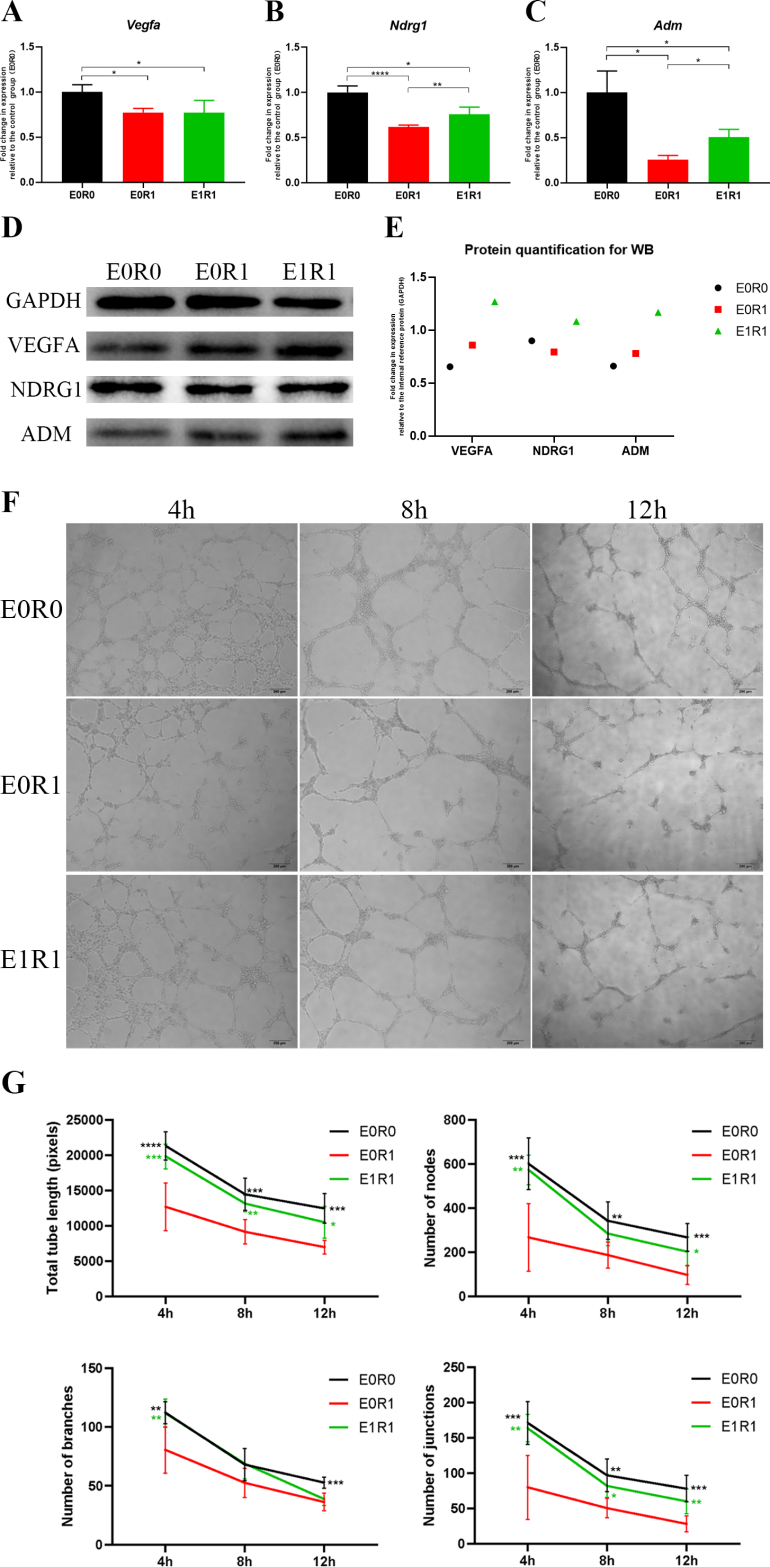
RP-Exos boosted pro-angiogenic gene and protein expression of macrophages, which were downregulated by IR. (A–C) RP-Exos upregulated expression of pro-angiogenic genes (*Vegfa, Ndrg1,* and *Adm*), reversing the side effect induced by IR. *n* = 4 per group (D) The protein levels of VEGFA, NDRG1, and ADM were analyzed by western blot. (E) Quantification analysis of the blots shown in (D). (F) HUVECs were cultured with conditioned medium collected from macrophages in the E0R0, E0R1, and E1R1 groups. E0R1 conditioned medium impaired tube formation ability of HUVECs, while tube sprouting results in the E1R1 conditioned medium-treated HUVEC group were closer to the normal condition (E0R0 group). (G) The number of nodes, junctions, branches, and total tube length were quantified. *n* = 6 per group. * = *P* < 0.05, ** = *P* < 0.01, *** = *P* < 0.001, **** = *P* < 0.0001.

As the KEGG and GO enrichment analyses show in Figs. 4C and 4E, genes related to “VEGF signaling pathway”, “HIF-1 signaling pathway”, and “positive regulation of angiogenesis” were downregulated in the E0R1 group compared to the E0R0 group. Meanwhile, expression of pro-angiogenic factors (VEGFA, ADM, and NDRG1) in macrophages were significantly downregulated after radiation (Figs. 5A–5C). Additionally, the HUVECs incubated in CM from macrophages receiving radiation displayed weaker tube formation ability than those receiving CM from normal macrophages (Figs. 5F, 5G). These results confirm that radiation was detrimental to macrophages in promoting angiogenesis.

### RP-Exos stimulated proliferation and M1 polarization in irradiated macrophages

RP-Exos promoted cell proliferation in irradiated macrophages, reversing the side effects of radiation (Fig. 3A). One possible mechanism behind this increased proliferation might be an increase in the number of cells in S phase (Figs. 3B, 3C–3K).

**Figure 6:**
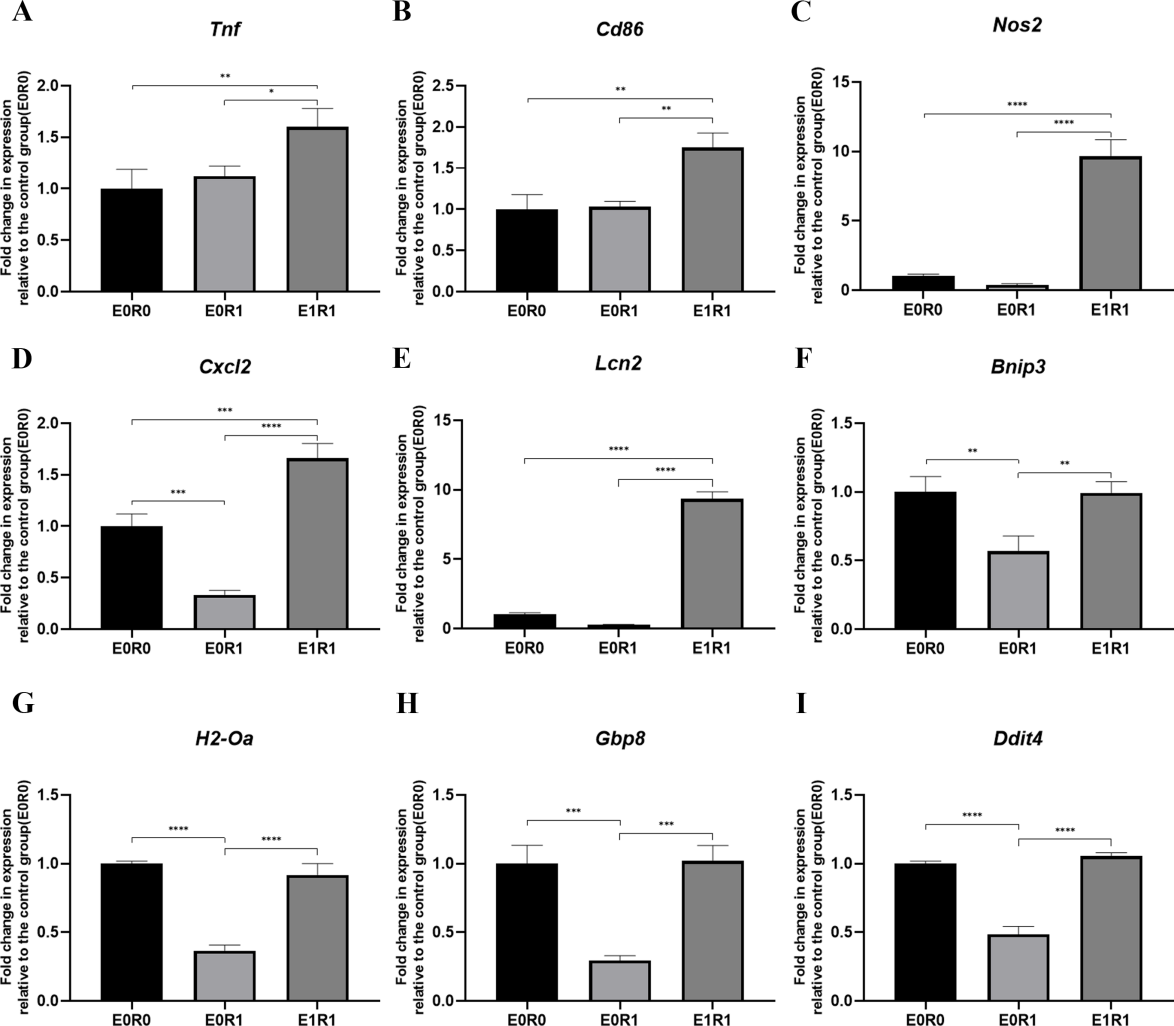
RP-Exos stimulated inflammatory response in irradiated macrophages. Inflammatory gene and M1 marker expression of (A) *Tnf,* (B) *Cd86,* (C) *Nos2*, (D) *Cxcl2*, (E) *Lcn2*, (F) *Bnip3*, (G) *H2-Oa*, (H) *Gbp8*, (I) *Ddit4* in E0R0, E0R1, and E1R1 groups. E0R1: macrophages receiving ironizing radiation. E1R1: macrophages receiving ionizing radiation and RP-Exo intervention. *n* = 3 per group. * = *P* < 0.05, ^∗∗^ = *P* < 0.01, ^∗∗∗^ = *P* < 0.001, ^∗∗∗∗^ = *P* < 0.0001.

As the KEGG enrichment analysis results show in Fig. 4D, classic inflammatory pathways such as “IL-17 signaling pathway” and “TNF signaling pathway” were activated by RP-Exos. In addition, upregulated DEGs were enriched for GO terms such as “immune response” and “chemokine-mediated signaling pathway” (Fig. 4F). Moreover, higher expression of M1-related genes such as *Tnf, Cd86,* and *Nos2* were observed in the E1R1 group than in the E0R1 group (Figs. 4G and 6). All of the above analyses suggest that RP-Exos rescued the reactivity of irradiated macrophages and promoted M1 polarization.

### RP-Exos promoted pro-angiogenic features in irradiated macrophages

RP-Exos reversed the deleterious effects of radiation on macrophages with respect to promoting angiogenesis. Upregulated genes in the E1R1 group compared with the E0R1 group were enriched for angiogenesis-related KEGG pathways and GO terms (Figs. 4D, 4F). In addition, RP-Exos rescued mRNA and protein expression of VEGFA, ADM, and NDRG1 in macrophages (Figs. 5A–5E). Furthermore, the CM from macrophages stimulated by RP-Exos promoted tube formation (Figs. 5F, 5G). These results indicate that RP-Exos can stimulate the pro-angiogenic potential of irradiated macrophages.

### RP-Exos favored angiogenesis and repair of skin and bone in irradiated rats

**Figure 7:**
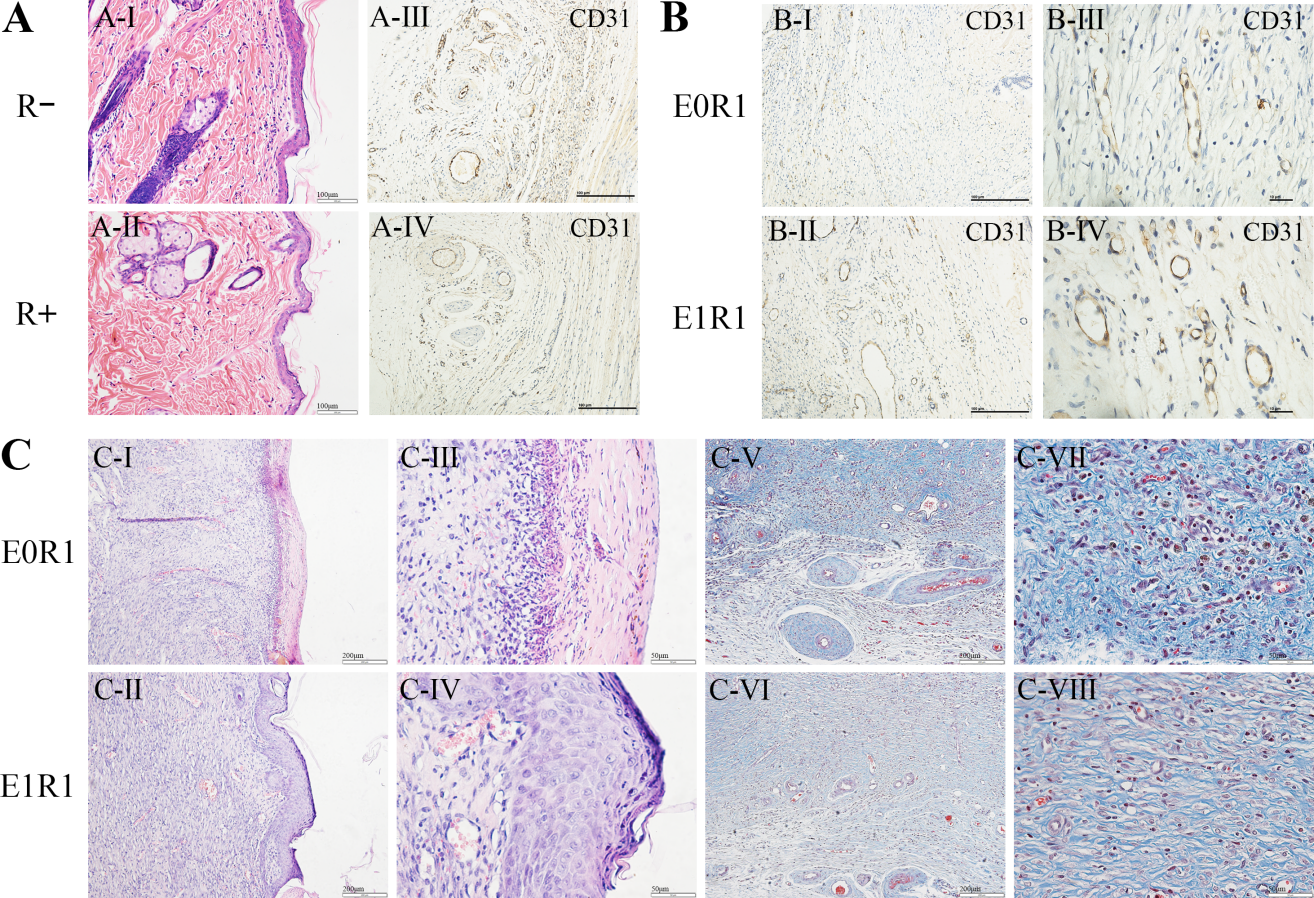
RP-Exos favored angiogenesis and regeneration in the skin defect model. (A) HE staining and CD31 immunohistochemical staining of untreated skin tissue sections in irradiated (R+) or non-irradiated (R-) rats. IR reduced the number of cells and blood vessels in skin tissues. (B, C) CD31 immunohistochemical staining, HE staining and Masson’s Trichrome staining of regenerated skin tissue in rats in the E0R1 and E1R1 groups at 21 days after operation. Tissues in the E1R1 group healed better and exhibited more blood vessels than tissues in the E0R1 group. E0R1: rats treated with PBS. E1R1: irradiated rats treated with RP-Exos.

**Figure 8:**
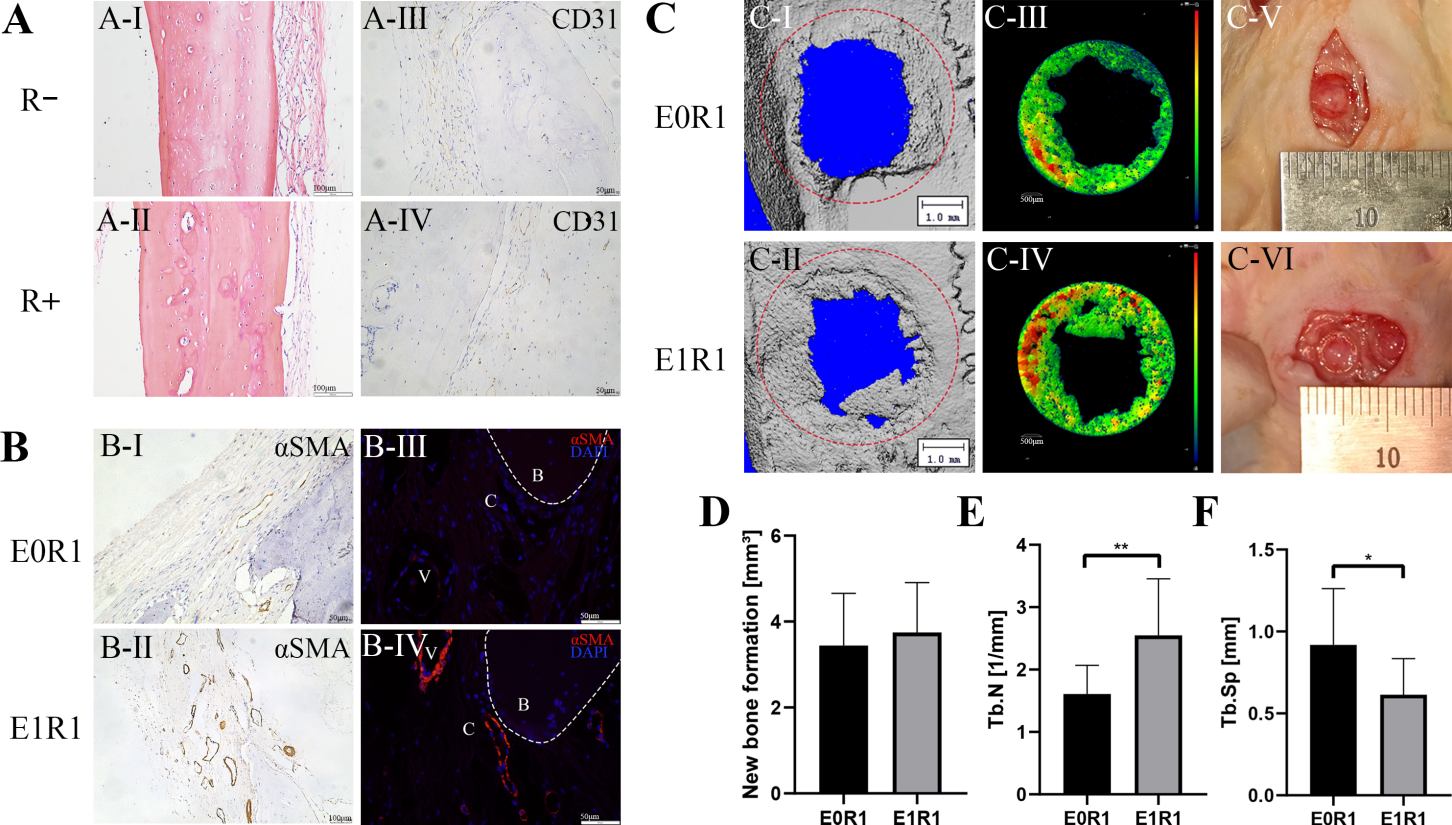
RP-Exos accelerated angiogenesis and regeneration in the bone defect model. (A) HE staining and CD31 immunohistochemical staining of untreated cranial bone in irradiated (R+) or non-irradiated (R-) rats. Cells and blood vessels were reduced by IR. (B) *α*SMA immunohistochemical and immunofluorescence staining of regenerated bone tissue in rats in the E0R1 and E1R1 groups. B: newly formed bone; V: blood vessels; C: connective tissues around newly formed bone. (C-I, II, III, IV) Representative micro-CT images of newly formed bone tissues. The red circle in (C-I, II) represents the defect ( five mm in diameter). Different colors in (C-III, IV) represent different bone densities, with red denoting denser and blue denoting sparser densities. (C-V, VI) Gross images of bone defect sites in the E0R1 and E1R1 groups. (D–F) Micro-CT quantitative analyses of new bone formation (mm ^3^), defined as bone within the red circle in (C-V,er); Tb.Sp (mm), trabecular separation; Tb.N (1/mm), trabecular number. *n* = 12 per group. * = *P* < 0.05, ** = *P* < 0.01.

Due to reproducibility, through-put and economic considerations, SD rats were popular animals in the field of regeneration (Spicer et al., 2012). The skin or calvarial defect model was established to detect the tissue regenerative effect of RP-Exos in vivo. No animal was excluded. As Figs. 7A and 8A reveal, both cell and blood vessel numbers in skin or bone tissue were reduced following radiation treatment, revealing the side effect of radiation on tissues.

In the E1R1 group, mature epidermal layers were observed, with clear basal layers, spiny layers, granular layers, strata lucidum, and strata corneum (Figs. 7C-II, IV). However, healing of the epidermal layer was delayed in the E0R1 group, as evidenced by the lack of cell structure formation (Figs. 7C-I, III). The collagen fibers in the E0R1 group were disordered and scattered (Figs. 7C-V, VII), while in the E1R1 group, collagen fibers were arranged in an orderly manner (Figs. 7C-VI, VIII). In the meantime, more vascular endothelial cell-specific markers (Fig. 7B) were detected in the E1R1 group. Thus, it is reasonable to infer that RP-Exos promote skin regeneration by driving the angiogenesis process.

In the bone defect models, micro-CT analysis demonstrated a significant increase in trabecular number and a decrease in trabecular separation in the E1R1 group (Figs. 8D–8F, raw data are available as a Supplemental File), which suggested that RP-Exos rescued radiation-induced osteoporosis. In addition, higher expression of vascular markers (anti-*α*SMA) in the newly formed bone was observed in the E1R1 group (Fig. 8B), indicating that RP-Exos resulted in increased density of trabecular bone, possibly through the promotion of angiogenesis.

## Discussion

Because of their remarkable capacity for intercellular communication, exosomes are attracting increased attention in the field of tissue repair (Meldolesi, 2018; Toh et al., 2018; Zhu et al., 2020). Recent research reports that exosomes derived from human umbilical cord blood plasma can stimulate neovascularization and accelerate the wound healing process, thereby exhibiting great potential for plasma exosomes in boosting regeneration (Hu et al., 2018b). In this study, we isolated exosomes from whole blood plasma, which is more accessible than umbilical cord blood plasma. First, we confirmed that plasma exosomes regulated the immune microenvironment by activating M1 polarization in irradiated macrophages, inducing angiogenic factors, and promoting vascular formation in vitro. Then, we successfully applied plasma exosomes to treat skin and bone defects in irradiated animals. Our research reveals the potential for plasma exosomes to be used as immunomodulatory agents with angiogenic capacity to treat radiation-associated vascular disorders and facilitate tissue repair (Fig. 1).

### Effect of IR on macrophages

Radiotherapy is the most common treatment for patients with head and neck cancer (Caudell et al., 2017). However, it is a double-edged sword, downregulating angiogenesis and interrupting normal wound healing and bone defect repair. Numerous researchers have investigated radiation-associated vascular dysfunction and found that radiation causes endothelial cell apoptosis (Paris et al., 2001), promotes structural disorganization and detachment of the aortic endothelium (Hauer-Jensen, Fink & Wang, 2004), and enhances vascular permeability (Hamada et al., 2020). However, the role the immune system plays in damaging the blood supply is rarely considered. Our study shows that radiation inhibited the cell proliferation of macrophages by inducing G2/M phase arrest and weakened the immune response. More importantly, we found that the pro-angiogenic capability of macrophages was decreased after receiving radiation treatment, attaching importance to the immune microenvironment in the context of radiation-induced adverse vascular effects.

### RP-Exos exhibited immunomodulatory capacities by promoting M1 polarization

Macrophages can polarize into several phenotypes, basically divided into M1 and M2 groups. M1 macrophages are classically activated and pro-inflammatory, whereas M2 macrophages are alternatively activated and are closely related to wound healing (Funes et al., 2018). Our results demonstrate that RP-Exos enhanced M1 polarization and led to a more inflammatory microenvironment.

The underlying mechanism could be related to the toll-like receptor (TLR) signaling pathway. In this study, TLR signaling and TNF were upregulated after RP-Exos treatment. Thus, it is possible that RP-Exos acted as ligands, stimulating the TLR signaling pathway, which further induced the production of TNF (Fortier, 2001). TNF, in turn, activated M1 macrophages in an autocrine fashion, enhancing M1 polarization (Fortier, 2001).

Exosome regulation of macrophage polarization has recently attracted much attention. Tubular epithelial cell-derived exosomes mediate M1 macrophage activation through miR-19b-3p in the case of tubulointerstitial inflammation (Lv et al., 2020). A systematic review concluded that exosomes of various sources could promote M2 macrophage polarization (Khalaj et al., 2020). Plasma exosomes of rats undergoing circadian rhythm disruptions caused increased M1/M2 ratios (Khalyfa et al., 2017). All of the above confirmed the potential of exosomes to modulate macrophage polarization.

### RP-Exos benefited angiogenesis, which was associated with their immunoregulatory functions

In our study, VEGFA, NDRG1, and ADM were upregulated by RP-Exos in macrophages. VEGFA plays an important role in angiogenesis, especially in the initiation of new capillary formation (Risau, 1997). Stimulated by VEGFA, endothelial cells differentiate into tip cells and stalk cells, which lead and support sprouting vessels, respectively (Spiller et al., 2014). NDRG1 facilitates VEGFA-induced angiogenesis through PLCγ1/ERK signaling (Watari et al., 2020; Watari et al., 2016). ADM upregulates the expression of VEGF (Iimuro et al., 2004). All of these factors contribute to better blood vessel formation, in accordance with our in vitro and in vivo results.

Prior work has demonstrated that macrophages play a critical role throughout the duration of vessel formation and anastomosis (Gurevich et al., 2018). Once injury occurs, macrophages are among the first to sense the injury and accumulate at the site where regeneration is needed, contributing to vessel tip sprouting and enhancing the recovery of blood flow (Hong & Tian, 2020). For a long time, M2 rather than M1 macrophages have been considered pro-angiogenic (Takeda et al., 2011). However, in this study, M1 polarization induced by RP-Exos also provided a suitable microenvironment for vascular remodeling.

TNF, CXCL2, and LCN2 are typical inflammatory factors and M1 markers secreted by macrophages. They also facilitate the process of angiogenesis. A number of studies have examined the positive role of TNF on angiogenesis. TNF was reported to induce formation of capillary blood vessels in the rat cornea and the developing chick chorioallantoic membrane (Leibovich et al., 1987). TNF primes endothelial cells for angiogenic sprouting by inducing a tip cell phenotype (Sainson et al., 2008). Reduced angiogenesis can be observed in TNFR2-KO mice (Luo et al., 2006). TNF induces VEGFR2-Etk association and reciprocal activation, which leads to Akt activation, further contributing to endothelial cell migration and angiogenesis (Zhang et al., 2003). In addition, TNF positively modulates arteriogenesis, likely via signaling through its p55 receptor (Hoefer et al., 2002). TNF can also upregulate VEGFR2 expression (Giraudo et al., 1998). CXCL2 also has a role during angiogenesis (Hardaway et al., 2015; Palacios-Arreola et al., 2014; Strieter et al., 2006). The CXCL2/CXCR2 pathway activates HIF-1α and VEGF (Bodnar, 2015). Overexpression of CXCL2 promotes HUVEC tube formation. LCN2 can also enhance VEGF-induced angiogenesis (Yang et al., 2013) by inducing the production of HIF-1α and VEGF (Hu et al., 2018a). Downregulation of LCN2 leads to suppressed angiogenesis (Guo et al., 2016). Corroborative findings similar to our results have also been reported: human endothelial cells form more vessels after exposure to M1 macrophages (Graney et al., 2020) and dampening of TNF expression in macrophages leads to impaired neoangiogenesis (Gurevich et al., 2018). All of the above evidence confirms that RP-Exos foster a favorable immune microenvironment for angiogenesis, further promoting the formation of blood vessel and tissue repair.

Although this study demonstrated that RP-Exos are effective in pro-angiogenesis and useful in rescuing poor vascularization due to radiotherapy, we acknowledge that there are still some limitations to our research that need to be explored further in future investigations. First, the exosomes extracted using Exoquick Exosome Isolation Kit might contain some heteroproteins from plasma. Second, the RAW 264.7 cell line was chosen rather than crude primary cultured macrophages because cell lines provide a more uniform reaction and facilitate the replication of our results by other researchers. However, primary cells are more representative of the true activity in vivo. In addition, the precise mechanisms by which RP-Exos influence macrophages are not fully understood. It also remains unclear what RP-Exos component is contributing to its effect on macrophages. However, because plasma exosomes are easily accessible and have high biocompatibility, we stress the potential for plasma exosomes to function as novel immunomodulators with the ability to accelerate regeneration in irradiated tissue by promoting angiogenesis.

## Conclusions

In this study, we have investigated the application of exosomes derived from rat plasma to rescue ionizing radiation-induced low blood supply damage through mediating the immune microenvironment. The results showed that RP-Exos contributed to neovascularization and tissue regeneration through activation of inflammation and upregulate the expression of pro-angiogenesis factors such as VEGFA, NDRG1, and ADM in macrophages. Using irradiated bone and skin defect models, we further demonstrated that RP-Exos showed excellent potency in increasing vessel formation and rescuing radiation-impaired tissue regeneration. This study offers a perspective for the application of exosomes derived from plasma to be novel immune regulators for the promotion of angiogenesis.

##  Supplemental Information

10.7717/peerj.11147/supp-1 Supplemental Information 1 Raw data of CCK-8 analysis, cell cycle analysis, PCR analysis, tube formation assay, WB quantification and micro-CT analysis

10.7717/peerj.11147/supp-2 Supplemental Information 2 Primers used for quantitative RT-PCR

10.7717/peerj.11147/supp-3 Supplemental Information 3 Western blot analysis of CD63 on RP-Exos and supernatant

10.7717/peerj.11147/supp-4 Supplemental Information 4 Western blot analysis of TSG101 on RP-Exos and supernatant

10.7717/peerj.11147/supp-5 Supplemental Information 5 Western blot analysis of the protein levels of ADM

10.7717/peerj.11147/supp-6 Supplemental Information 6 Western blot analysis of the protein levels of GAP

10.7717/peerj.11147/supp-7 Supplemental Information 7 Western blot analysis of the protein levels of NDRG1

10.7717/peerj.11147/supp-8 Supplemental Information 8 Western blot analysis of the protein levels of VEGFA

10.7717/peerj.11147/supp-9 Supplemental Information 9 Completed author checklist

## Additional Information and Declarations

### DNA Deposition

The following information was supplied regarding the deposition of DNA sequences: Data are available at the SRA database: SRR13209741 to SRR13209749.

### Data Availability

The following information was supplied regarding data availability: Raw data and uncropped western blot images are available in the Supplemental Files.
