# Supplementary material for: Exosomes derived from plasma: promising immunomodulatory agents for promoting angiogenesis to treat radiation-induced vascular dysfunction
Source: PeerJ. 2021 Apr 2;9:e11147. doi: 10.7717/peerj.11147 (PMC8020864; doi:10.7717/peerj.11147)
Supplement: Supplemental Information 2 [file peerj-09-11147-s002.docx]

Table S1. **Primers used for quantitative RT-PCR.**

| Gene name | Forward | Reverse |
| --- | --- | --- |
| *Adm* | GAAAGAAGTGGAATAAGTGGGC | GATCAAGAGTCTGGGTAGGAAC |
| *Bnip3* | ATGGCAATGGGAGCAGCGTTC | TGGTGTCTGGGAGCGAGGTG |
| *Cd86* | ACCAGGCTCTACGACTTCACAATG | TCACTGAAGTTGGCGATCACTGAG |
| *Cxcl2* | GGTTGACTTCAAGAACATCCAG | TTGAGAGTGGCTATGACTTCTG |
| *Ddit4* | TAAGTTCTGCCAACTCTTCCTT | CGGAGCTGTAGAGTTTCTTCTT |
| *Gbp8* | CTTCATGGAGCATTCCTTCAAG | TTCAACAGGAAAAGCACTTTCG |
| *H2-Oa* | CTTCTACTCTCAGCCTAACCAC | AGATCAGGGTCTCTATGGTGTC |
| *Lcn2* | CGCTACTGGATCAGAACATTTG | CTTGCACATTGTAGCTCTGTAC |
| *Ndrg1* | CTTATTCATCAGCGCCTACAAC | TCTTCGTTGGGTCCAATTTAGA |
| *Nos2* | GAGACGCACAGGCAGAGGTTG | CAGGAAGGCAGCAGGCACAC |
| *Tnf* | GCCAACGGCATGGATCTCAA | TAGCAAATCGGCTGACGGTG |
| *Vegfa* | GGATCAAACCTCACCAAAGCC | TGTTCTGTCTTTCTTTGGTCTGC |
