# Supplementary figures and images for: Exosomes derived from plasma: promising immunomodulatory agents for promoting angiogenesis to treat radiation-induced vascular dysfunction
Source: PeerJ. 2021 Apr 2;9:e11147. doi: 10.7717/peerj.11147 (PMC8020864; doi:10.7717/peerj.11147)

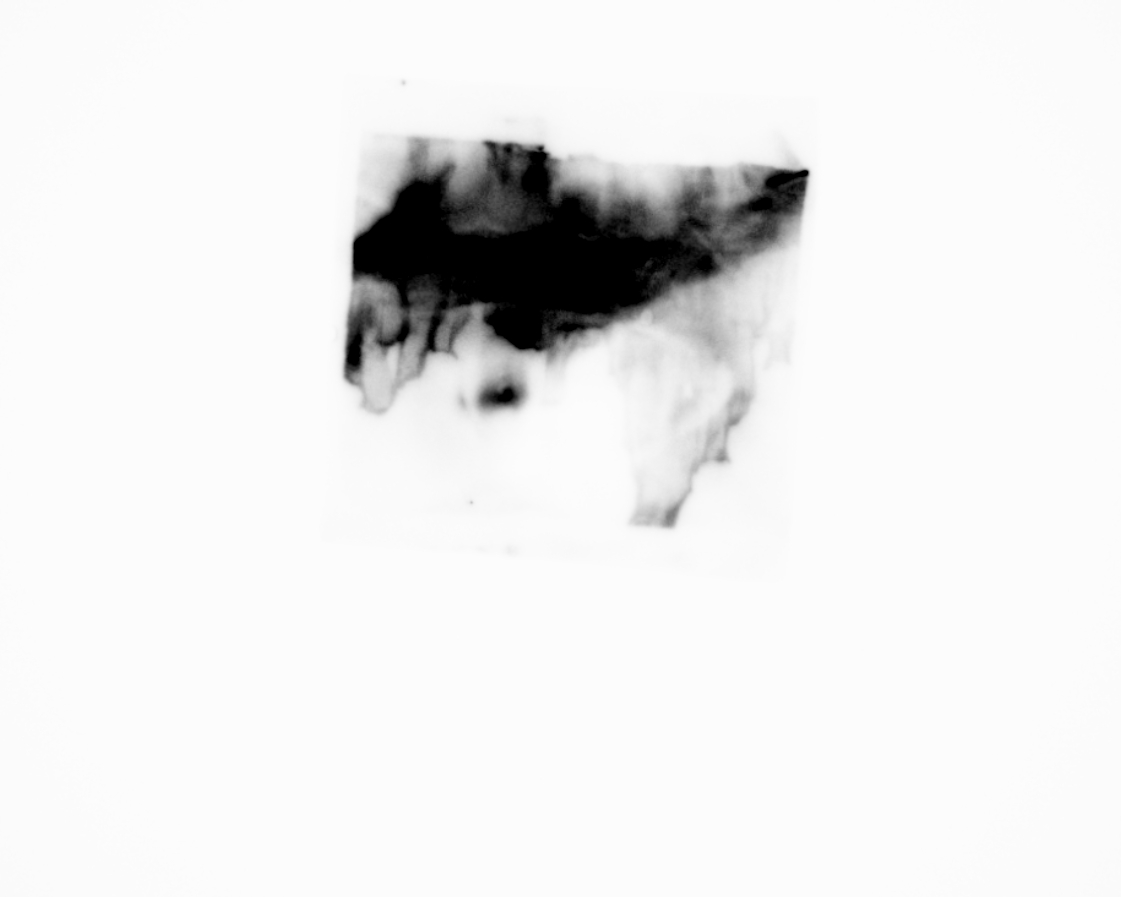

Supplement: Supplemental Information 3 [file peerj-09-11147-s003.jpg]

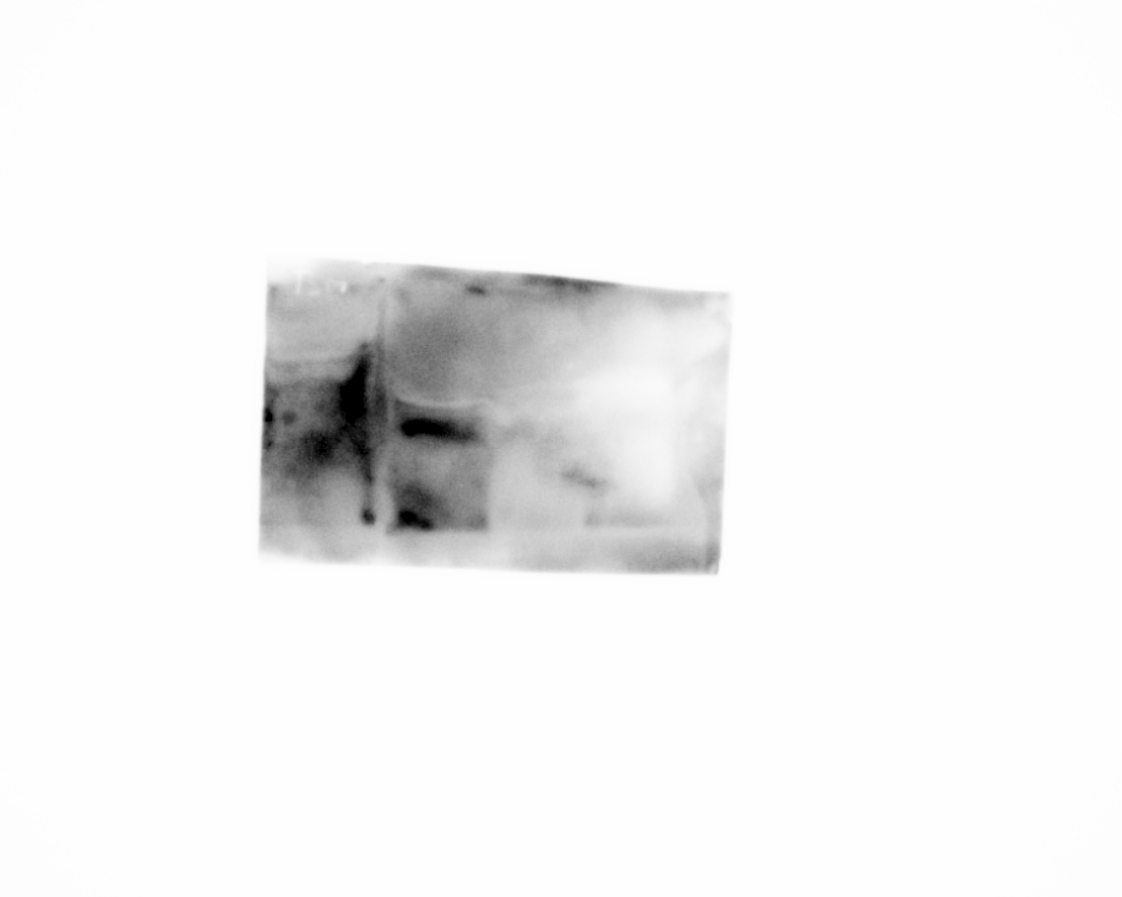

Supplement: Supplemental Information 4 [file peerj-09-11147-s004.jpg]

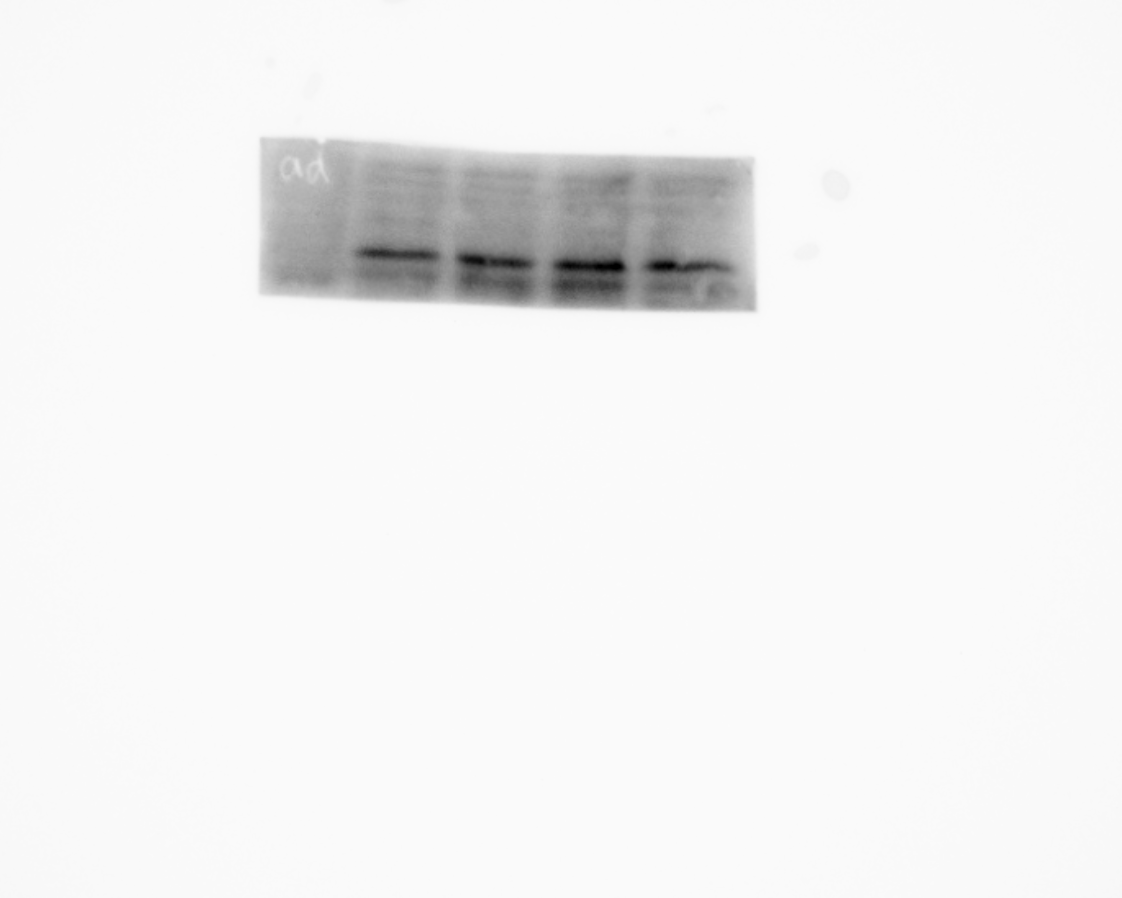

Supplement: Supplemental Information 5 [file peerj-09-11147-s005.png]

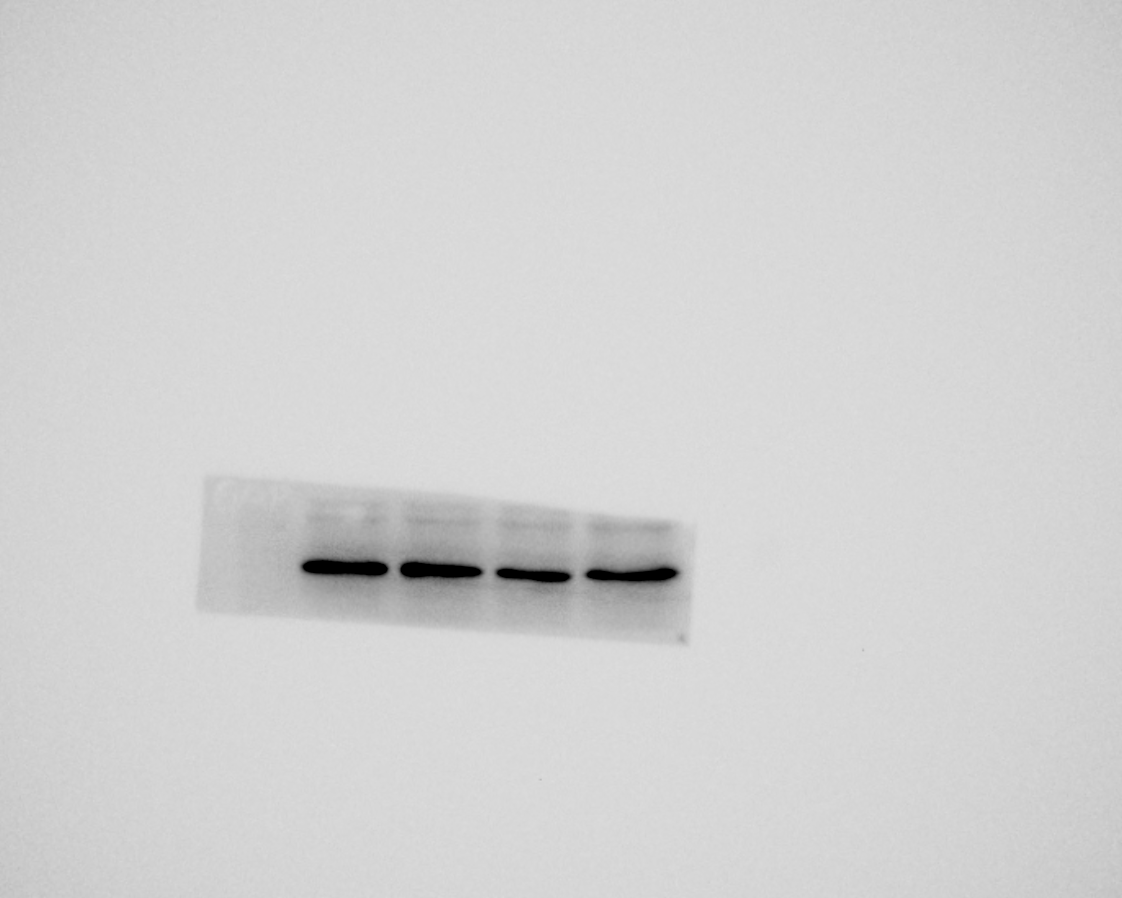

Supplement: Supplemental Information 6 [file peerj-09-11147-s006.png]

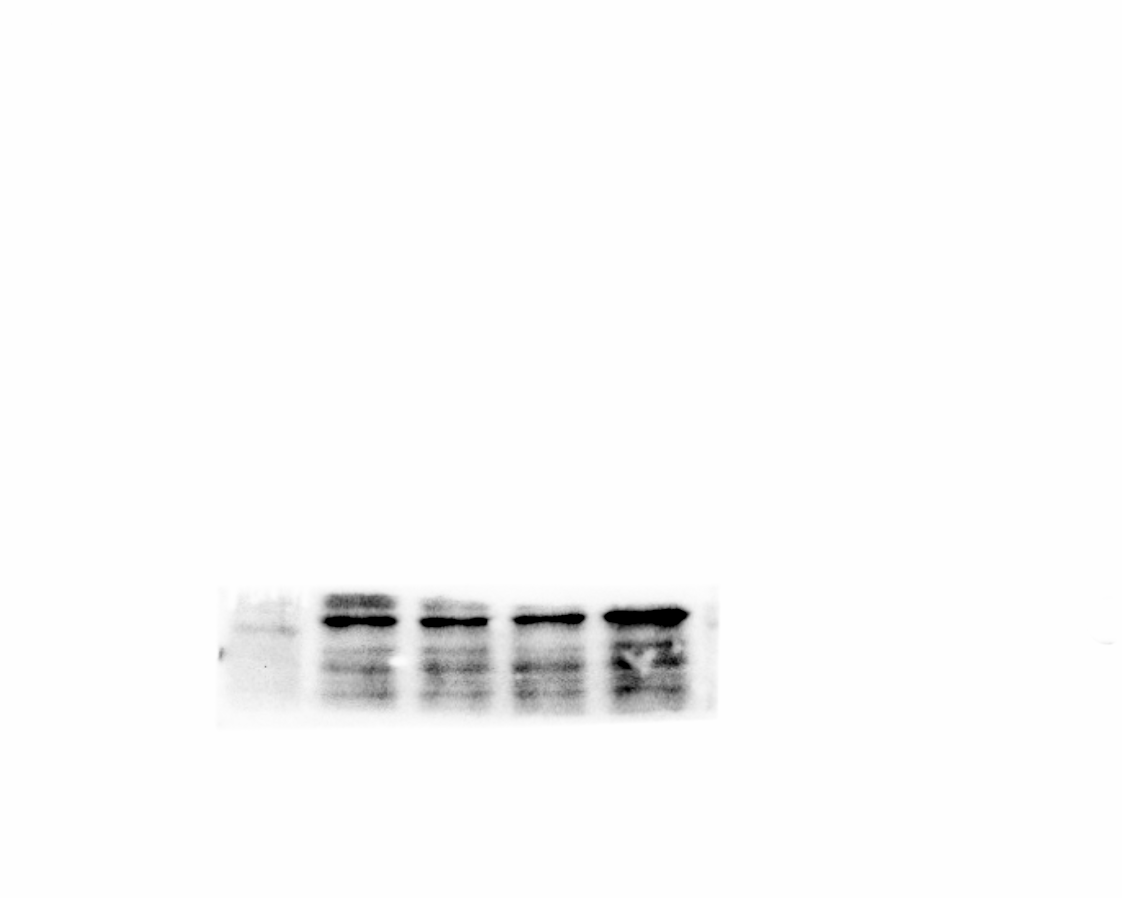

Supplement: Supplemental Information 7 [file peerj-09-11147-s007.png]

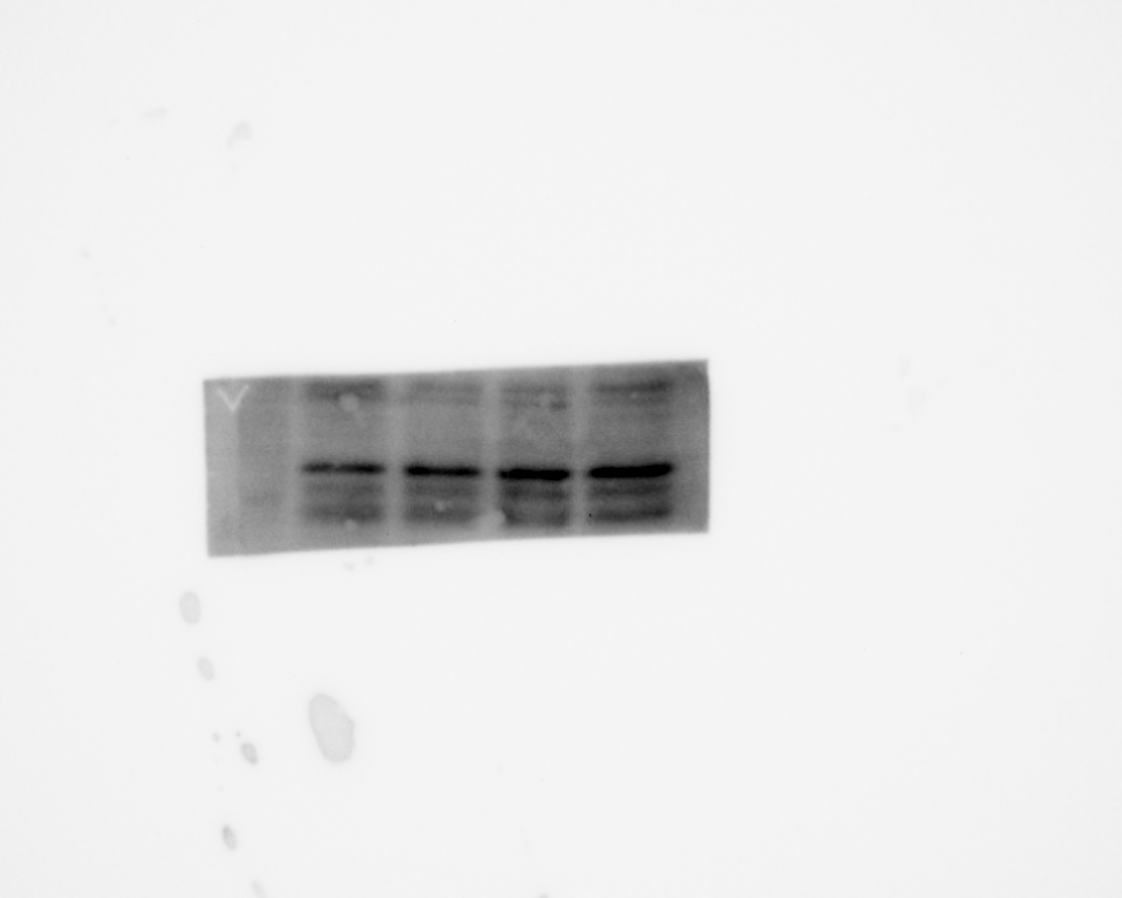

Supplement: Supplemental Information 8 [file peerj-09-11147-s008.png]
